# *Vigna pandeyana* (Fabaceae), a new species from northern Western Ghats, India

**DOI:** 10.3897/BDJ.3.e4606

**Published:** 2015-03-06

**Authors:** Sayajirao Gaikwad, Ramchandra Gore, Sonali Randive

**Affiliations:** ‡ Life Science Research Laboratory, Walchand College of Arts and Science, Solapur- 413 006, Maharashtra, India

**Keywords:** Taxonomy, *
Ceratotropis
*, dimorphic shoots, cleistogamous flowers.

## Abstract

**Background:**

**Vigna** subg. *Ceratotropis* (Piper) Verdc. represents a homogenous and distinct group of species with highly specialized complex floral characters. It is most diverse in Asia. India, with 24 species, represents a secondary center of species diversity of the subgenus.

**New information:**

A new species, *Vigna pandeyana* RD Gore, SP Gaikwad & SD Randive, is described from hill slopes of the northern Western Ghats of India. It resembles *Vigna yadavii* Gaikwad et al. and *Vigna dalzelliana* (Kuntze) Verdc. but differs from the latter in its dimorphic shoots (some subterranean, with cleistogamous flowers) and densely hairy pods, from the former by its curved style, flattened style beak, foveolate seed coat and absence of standard protuberance and horn-like keel pocket in cleistogamous flowers.

## Introduction

The pantropical genus *Vigna* Savi of the tribe Phaseoleae comprises about 90 species distributed in six subgenera (Thulin et al. 2004, Delgado-Salinas et al. 2011, Toomoka et al. 2010). Vigna subg. Ceratotropis (Piper) Verdc. has its center of species diversity in Asia and is popularly known as Asian *Vigna* (Tomooka et al. 2002b). Earlier, it was represented by 22 species in three sections, viz. *V.* sect. *Aconitifoliae* Tomooka & Maxted, sect. *Angulares* Tomooka & Maxted and sect. *Ceratotropis* (Piper) Verdc. (Tomooka et al. 2002a, Toomoka et al. 2010). Moreover, *V. sahyadriana* Aitawade et al. and *V. yadavii* Gaikwad et al. have recently been described from India. Therefore, the number of species in the genus *Vigna* subgenus *Ceratotropis* is now 24.

In 2012, the authors came across an interesting species of *Vigna* on the hill slopes near Chalkewadi in Satara district of the Maharashtra State of India which has unusual dimorphic shoots and cleistogamous flowers. It was recollected in the subsequent two years for further studies of its vegetative and floral characters. Initially, the new species was confused with *Vigna yadavii* Gaikwad *et al*., as both hold cleistogamous flowers. However, a critical study of floral structures, seeds, seed coat and type of seed germination have revealed that it represents an undescribed species of *Vigna* subg. *Ceratotropis*. This has been confirmed by the perusal of relevant literature (Maréchal et al. 1978, Tateishi 1984, Babu et al. 1987, Tomooka et al. 2002a, Tomooka et al. 2002b, Maxted et al. 2004, Thulin et al. 2004, Lewis et al. 2005, Toomoka et al. 2010, Delgado-Salinas et al. 2011, Aitawade et al. 2012​ and Gaikwad et al. 2014) and experts' opinion on the identity of the species. The new species is described and illustrated in this paper.

## Taxon treatments

### Vigna pandeyana

RD Gore, SP Gaikwad & SD Randive, 2015 sp. nov.

urn:lsid:ipni.org:names:77145586-1

#### Materials

- **Type status:** Holotype. **Location:** continent: Asia; country: India; countryCode: IND; stateProvince: Maharashtra; county: Sahyadri Ranges; municipality: Satara district; locality: near Chalkewadi; verbatimElevation: 1050 m; verbatimLatitude: 17°35'45.9"N; verbatimLongitude: 73°50'39.3"E; verbatimCoordinateSystem: degrees minutes seconds; **Event:** eventDate: 27 September 2012; habitat: It grows in lateritic gravelly soil on hill slopes in or on grasses and herbs; fieldNumber: RD Gore 1007; fieldNotes: Twining annual herbs; stems terete; flowers are of two kinds; the chasmogamous flowers present on leafy aerial shoots while cleistogamous flowers present on leafless shoots, which are close to soil surface; pods hairy; **Record Level:** language: English; institutionID: CAL; basisOfRecord: Herbarium specimen
- **Type status:** Isotype. **Location:** continent: Asia; country: India; countryCode: IND; stateProvince: Maharashtra; county: Sahyadri Ranges; municipality: Satara district; locality: near Chalkewadi; verbatimElevation: 1050 m; verbatimLatitude: 17°35'45.9"N; verbatimLongitude: 73°50'39.3"E; verbatimCoordinateSystem: degrees minutes seconds; **Event:** eventDate: 27 September 2012; habitat: It grows in lateritic gravelly soil on hill slopes in or on grasses and herbs; fieldNumber: RD Gore 1007; fieldNotes: Twining annual herbs; stems terete; flowers are of two kinds; the chasmogamous flowers present on leafy aerial shoots while cleistogamous flowers present on leafless shoots, which are close to soil surface; pods hairy; **Record Level:** language: English; institutionID: BSI, K, SUK; basisOfRecord: Herbarium specimen
- **Type status:** Other material. **Location:** continent: Asia; country: India; countryCode: IND; stateProvince: Maharashtra; municipality: Nasik District; locality: Kasara-Ghat near Igatpuri; verbatimElevation: 365 m; verbatimLatitude: 19°41'02.1"N; verbatimLongitude: 73°29'58.3"E; **Event:** eventDate: 10-11-2012; fieldNumber: RD Gore 1042; fieldNotes: Twining herbs; leaves stipulate; stipules submedifixed; chasmogamous flowers yellow & Cleistogamous flowers white; pods falcate to straight; seeds well developed; **Record Level:** language: English; institutionID: Botanical Survey of India, Calcutta (CAL).
- **Type status:** Other material. **Location:** continent: Asia; country: India; countryCode: IND; stateProvince: Maharashtra; municipality: Nasik District; locality: Kasara-Ghat near Igatpuri; verbatimElevation: 365 m; verbatimLatitude: 19°41'02.1"N; verbatimLongitude: 73°29'58.3"E; **Event:** eventDate: 10-11-2012; fieldNumber: *RD Gore* 1042a; fieldNotes: Twining herbs; leaves stipulate; stipules submedifixed; chasmogamous flowers yellow & Cleistogamous flowers white; pods falcate to straight; seeds well developed; **Record Level:** language: English; institutionID: Botanical Survey of India, Pune (BSI).
- **Type status:** Other material. **Location:** continent: Asia; country: India; countryCode: IND; stateProvince: Maharashtra; municipality: Nasik District; locality: Kasara-Ghat near Igatpuri; **Identification:** identifiedBy: SP Gaikwad & RD Gore; **Event:** eventDate: 10-11-2012; fieldNumber: *SD Randive* 322; fieldNotes: Twining herbs; flowers yellow; pods slightly hairy; **Record Level:** institutionID: Herbarium of Walchand College of Arts & Science, Solapur (WCAS).
- **Type status:** Other material. **Location:** continent: Asia; country: India; countryCode: IND; stateProvince: Maharashtra; municipality: Nasik District; locality: Saptshrungi hills (Kalvan tehsil); **Identification:** identifiedBy: SP Gaikwad & RD Gore; **Event:** eventDate: 9-11-2012; fieldNumber: RD Gore 1040; fieldNotes: Twining herbs; flowers both chasmogamous (yellow) and cleistogamous (white/albino); **Record Level:** institutionID: Herbarium of Walchand College of Arts & Science, Solapur (WCAS).
- **Type status:** Other material. **Location:** continent: Asia; country: India; countryCode: IND; stateProvince: Maharashtra; municipality: Satara District; locality: Pasarnighat; **Identification:** identifiedBy: MM Aitawade; **Event:** eventDate: 21-10-2011; fieldNumber: *SP Sutar* 156; fieldNotes: Closely resembles with *Vigna silvestris* but differs in pod & seed number; seeds rectangular; **Record Level:** institutionID: Kew herbarium (K); collectionCode: K000978011; source: http://apps.kew.org/herbcat/getImage.do?imageBarcode=K000978011
- **Type status:** Other material. **Location:** continent: Asia; country: India; countryCode: IND; stateProvince: Maharashtra; municipality: Satara District; locality: Pasarnighat; **Event:** eventDate: 21-10-2011; fieldNumber: *SP Sutar* 156; fieldNotes: Closely resembles with *Vigna silvestris* but differs in pod & seed number; seeds rectangular; **Record Level:** institutionID: Herbarium of Shivaji University, Kolhapur (SUK).
- **Type status:** Other material. **Location:** continent: Asia; country: India; countryCode: IND; stateProvince: Maharashtra; municipality: Sangli; locality: Dandoba hills; **Event:** eventDate: 28-09-1989; fieldNumber: *AN Londhe* 170037; **Record Level:** institutionID: Botanical Survey of India, Pune (BSI).

#### Description

**Figure 1.**
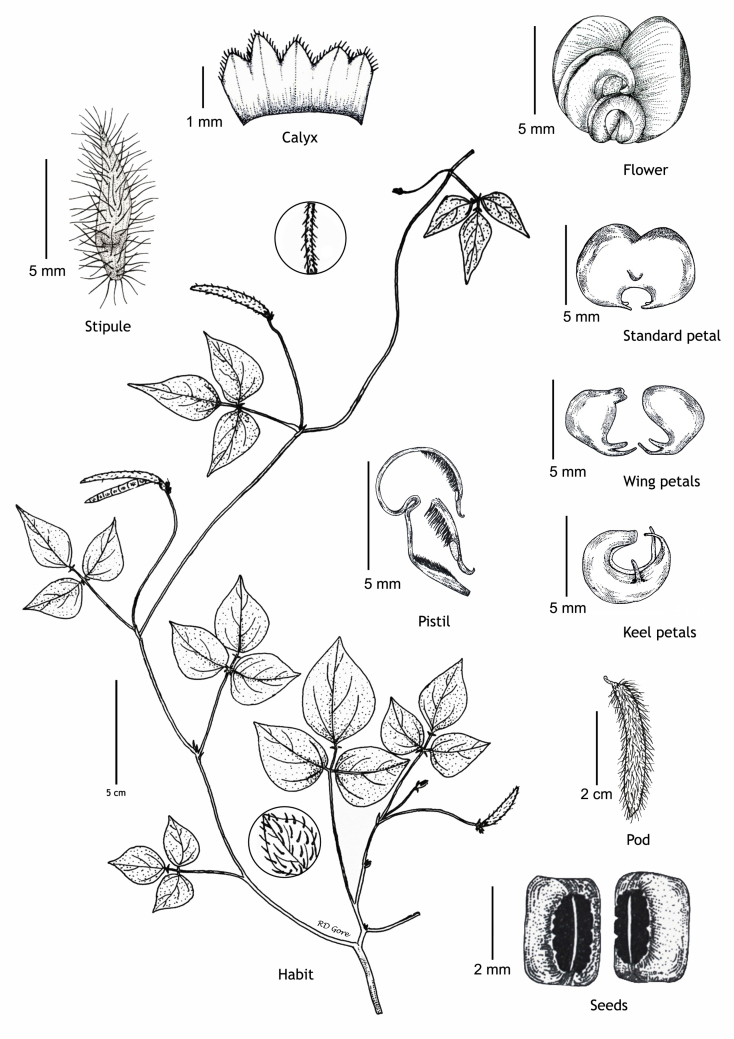
Details of chasmogamous flowers of *Vigna pandeyana* Gore et al. (all from *RD Gore* 1007, drawn by Ramchandra D. Gore).

**Figure 2.**
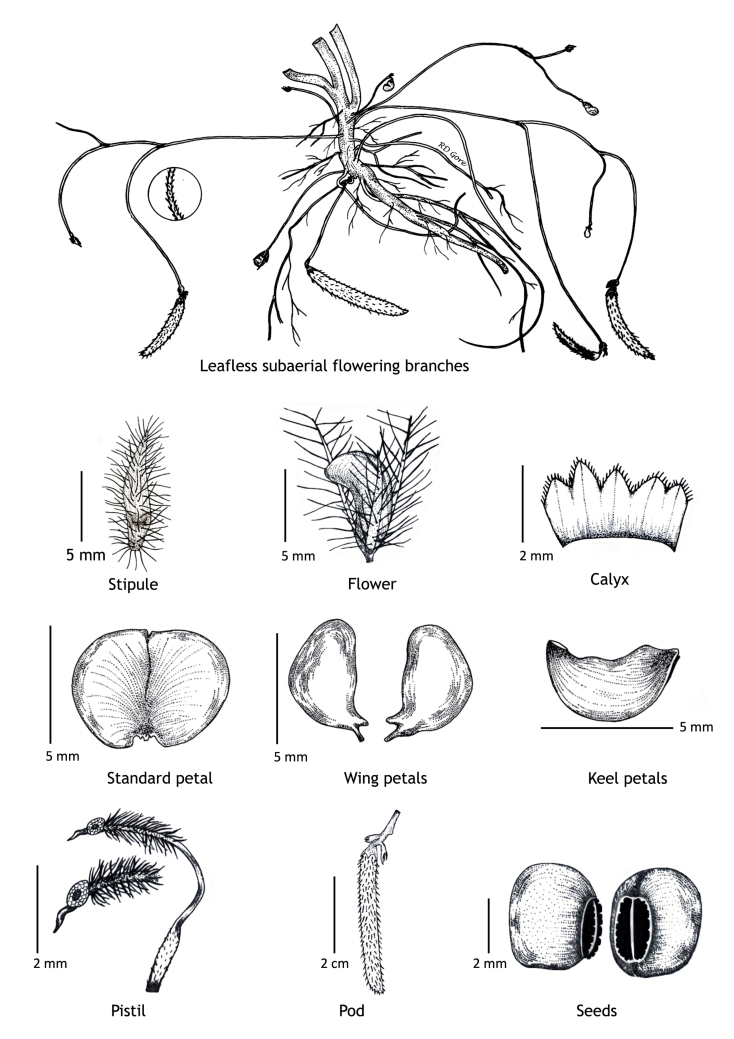
Details of cleistogamous flowers of *Vigna pandeyana* Gore et al. (all from *RD Gore* 1007, drawn by Ramchandra D. Gore).

**Figure 7.**
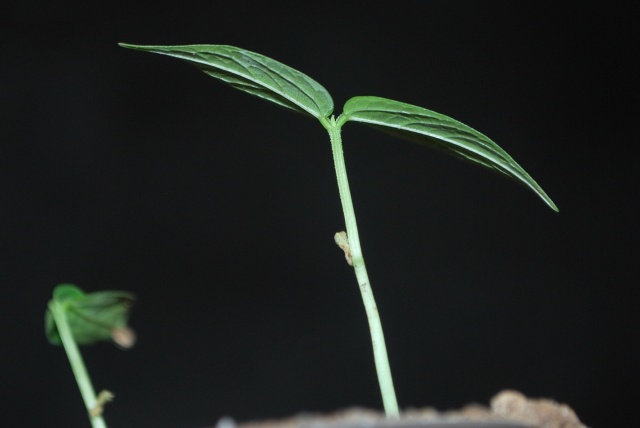
Seed germination of *Vigna pandeyana* Gore et al. (photograph by Gore RD).

Twining annual herbs. Stems terete, up to 2 m long, covered with 1-3 mm long, retrorse or spreading bulbous based hairs. Leaves 3-foliolate; petioles 3-5(-7) cm long, densely covered with retrorse or spreading bulbous based hairs; stipules elliptically lanceolate, medifixed, 7-9 mm long, 5-7 nerved, rounded at base, acute at apex, hairy on dorsal surface; stipels 2, linear-lanceolate, 2-3 mm long, acute or acuminate at apex, glabrous. Leaflets membranous, entire; lateral ones obliquely ovate or rhomboid to lanceolate, 2.2-5.5 x 1.5-2.3 cm, obliquely rounded at base, acute or shortly acuminate at apex, sparsely hairy; terminal leaflet ovate or rhomboid-lanceolate, 3-6.4 x 1.2-4 cm, base rounded (not oblique as in lateral leaflets), apex acute or shortly acuminate, sparsely hairy, margins entire or sometimes wavy; rachis 2-10 mm long, densely covered with whitish hairs. Flowers are of two kinds; the chasmogamous flowers present on leafy aerial shoots while cleistogamous flowers present on leafless subterranean shoots, which are close to soil surface. *Chasmogamous flowers*: yellow, 2-4 in axillary and terminal pseudo-racemes; peduncle 5-7.5 cm long, densely hairy with whitish-brown, 1-1.5 mm long, retrosely spreading hairs; pedicels 2-2.5 mm long, densely hairy as peduncle; bracts lanceolate, 2.5-3 x 0.8-1 mm, herbaceous, acute at apex, densely hairy; bracteoles 2, linear, 4-4.5 mm long, densely hairy. Calyx campanulate, *ca* 2 mm long; teeth triangular, 0.2-1.2 x 0.7-1 mm, sparsely hairy along margins. Standard petal yellow, asymmetrical, broadly ovate, 6-6.5 x 7.5-8.5 mm, emarginate at apex, central protuberance present inside (up to 0.9 mm long); claw *ca* 1 mm long. Right wing concealing the upper portion of the keel petals; claws 0.7-1 mm long; lamina obliquely obovate, 3.5-6.5 x 2.7-3.5 mm, notched at apex. Left wing claws 1-1.2 mm long; lamina obliquely obovate, 4-6.2 x 1.2-4 mm. Keel petals spirally incurved to left, 6-6.5 x 2.5-3.5 mm; horn-like pocket present (1.5-1.7 mm long) on the left side of keel petal. Stamens 9+1, included; staminal tube 4.5-5 mm long; free filament 8-9 mm long; anthers dorsifixed, 0.2-0.3 mm long. Pistil 1.5-1.8 cm long; ovary linear, 2.2-2.4 mm long, densely covered with long whitish hairs; style filiform, 1.2-1.3 cm long, ‘S’ shaped before stigma, beaked beyond stigma; beak 0.8-1 mm long, upwardly curved at apex. Pods ascending linear, cylindrical, 2-5 x 0.3-0.5 cm, straight or slightly curved, densely covered with brownish, 2-3 mm long spreading hairs, acute at apex. Seeds 4-10 per pod, broadly rectangular, 3-3.1 x 2-2.1 mm, brown, mottled with faint black patches, rounded or rectangular at both ends; seed coat porous with mesh-like reticulation; hilum well developed, rim-aril protruded, elliptic, 1.8-2 x 0.7-0.9 mm, whitish-brown. Germination epigeal; ecophylls petioles, simple and minutely pulvinous. *Cleistogamous flowers*: 2-5, whitish-yellow on leafless sub-aerial branches which produce on lower nodes of stem; pedicels 1-2 mm long; bracts lanceolate, 5-6 mm long, acute at apex, densely hairy; bracteoles 2, linear, 7-9 mm long, densely covered with 1-3 mm long bulbous based hairs. Calyx as in chasmogamous flowers. Standard petal symmetrical, broadly ovate, 4.5-5 x 5.5-6 mm, emarginate at apex, inside central protuberance absent; claws 0.3-0.5 mm long. Right wing petal slightly concealing the upper portion of the keel petal; claws 0.3-0.5 mm long; lamina obliquely ovate, 4.5-5 x 2-3 mm; auricle 0.1-0.2 mm long. Left wing petal spreading; claws 0.5-0.7 mm long; lamina ovate-elliptic, 4.9-5 x 2-2.7 mm, base oblique; auricle 0.3-0.5 mm long. Keel petals slightly curved, 4.8-5 x 1.5-2.5 mm. Stamens 9+1, dimorphic, out of 9 stamens in a bundle 4 short and 5 long; short stamens sterile with *ca* 1 mm long filaments, whereas long stamens fertile with *ca* 2 mm long filaments; staminal tube *ca* 1.5 mm long; free stamen sterile with *ca* 2 mm long filament; anthers dorsifixed 0.2-0.6 mm long. Pistil filiform, up to 6.5 mm long; ovary linear, 1.7-2 mm long, glabrescent or sparsely hairy; style filiform, 3-3.5 mm long, curved (not ‘S’ shaped), densely hairy before stigma with 0.4-0.6 mm long hairs, beaked beyond stigma; beak short, 0.3-0.5 mm long, straight, pointed at apex. Pods linear, cylindrical, straight, 2.5-4 x 0.3-0.4 cm, narrowed at both ends, sparsely hairy with short hairs (0.5 mm long), white hairs. Seeds 8-9 per pod, obliquely rounded, 3.5-4 x 3-3.5 mm, dark brown; seed coat foveolatewith mesh-like reticulation; hilum well developed, protruded, broadly elliptic, 2-2.2 x 0.4-0.7 mm, whitish. Germination epigeal; ecophylls petioles, simple and minutely pulvinous. (Figs 1, 2, 7​).

##### Flowering and fruiting

August-October.

#### Diagnosis

**Vigna yadavii** similis, ramis dimorphis, floribus cleistogamis vexillo sine processo carina sine marsupio corniformi, styli rostro applanato, seminum testa foveolata, hilo bene evoluto differt.

#### Etymology

The specific epithet honors Prof AK Pandey, Department of Botany, Delhi University, New Delhi (India), in recognition of his valuable contribution to the taxonomy of flowering plants of India.

#### Distribution

India, Maharashtra, Satara district, near Chalkewadi in Patan tahsil.

#### Ecology

It is a twining annual herb, grows on lateritic gravelly soil on hill slopes amonggrasses and herbs at about 1200 m altitude above mean sea level in Satara district of Maharashtra, India. The species has adapted to the monsoon seasonality. It thrives in humid climate with heavy rainfall during growth season. The seed germination takes place with onset of monsoon rain in the first week of June and the plant completes its life cycle with formation of seeds when rains ceasein mid October. The common associates of the species are *Carvia callosa* (Nees) Bremek., *Crotalaria nana* Burm. f., *Crotalaria vestita* Baker, *Cajanus lineatus* (Wight & Arn.) Maesen, *Eragrostis* spp., *Pseudarthria* spp., *Nogra dalzellii* (Baker) Merr. and *Themeda* spp.

#### Notes

Interestingly, two types of shoots are observed in the species, one a normal aerial leafy shoot and the other subterranean (close to soil surface) leafless shoot producedatthe lower nodes of the stem. The later shoots produce cleistogamous flowers, which remain closed. They show differences in the structure of their floral parts as compared to chasmogamous flowers such as standard petal without central protuberance inside, keel petals without horn-like pocket, curved style and short style beak (0.3-0.5 mm long). (Figs 3, 4, 5, 6​). The floral parts of chasmogamous flowers roughly correspond to the floral parts of the species of *Vigna* sect. *Ceratotropis* while cleistogamous floral parts correspond to the species of *V.* sect. *Aconitifoliae* of the same subgenus. Thus, it shows a combination of characters of species of both sections. The new species shows morphological similarities with *Vigna yadavii* Gaikwad et al. and *Vigna dalzelliana* (Kuntze) Verdc. but differs in its dimorphic branches, foveolate seed coat and absence of standard protuberance and horn-like keel pocket in cleistogamous flowers. A comparative account between above mentioned three species is given in Table 1.

## Supplementary Material

XML Treatment for Vigna pandeyana

## Figures and Tables

**Figure 3a.**
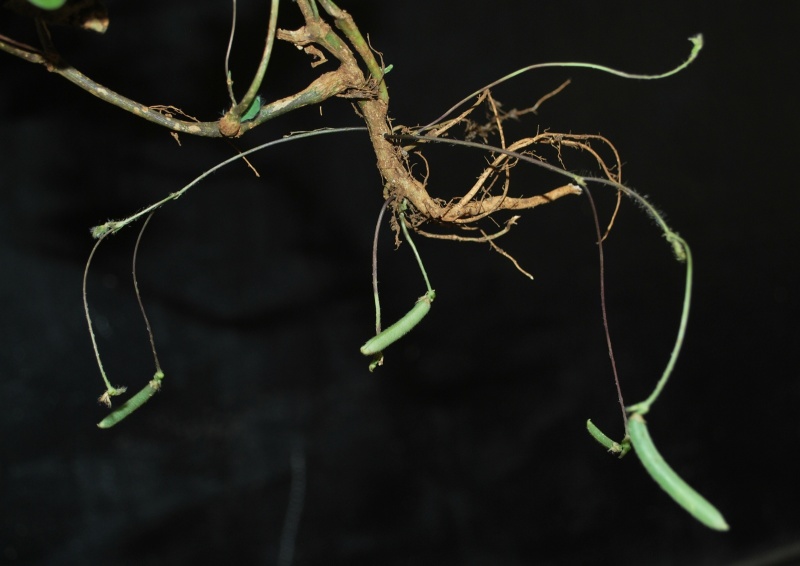
Leafless branches with pods

**Figure 3b.**
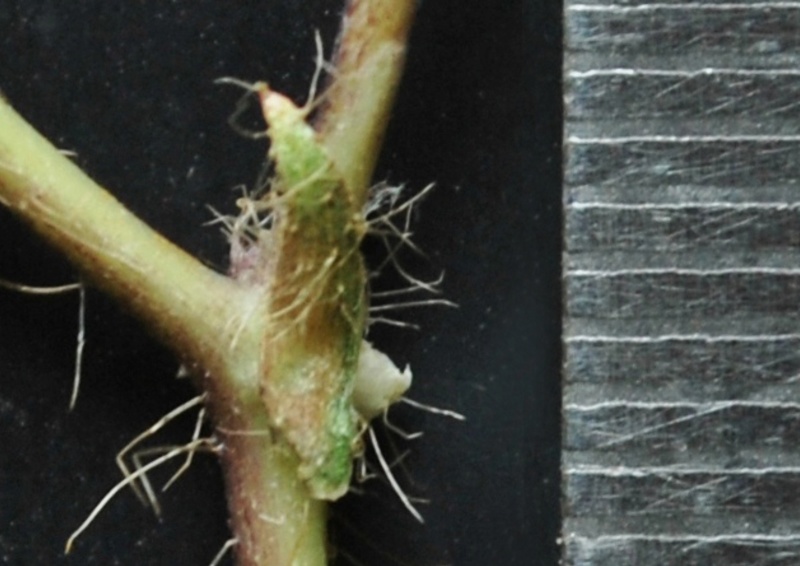
Stipule

**Figure 3c.**
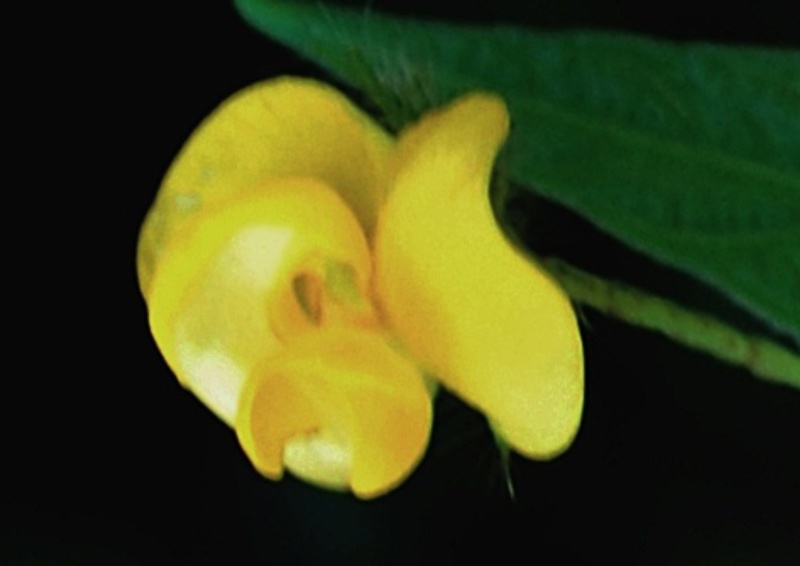
Chasmogamous flower

**Figure 3d.**
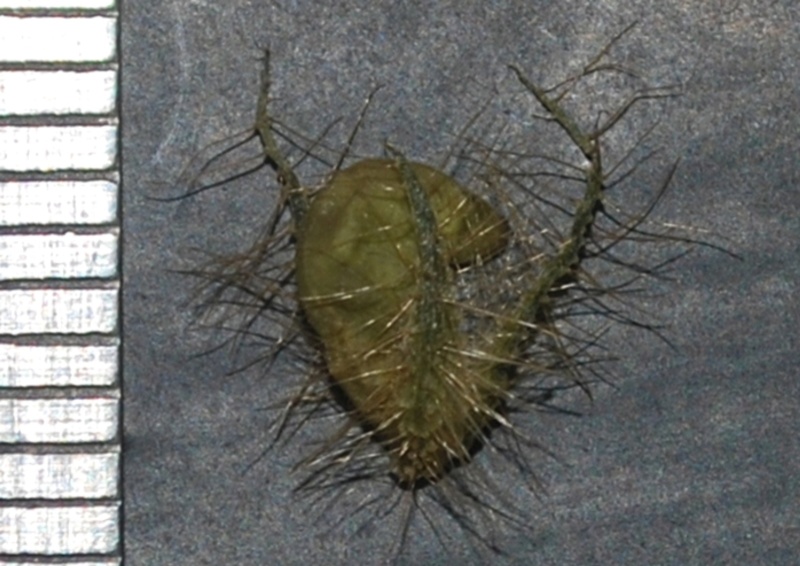
Cleistogamous flower with bracts and bracteoles

**Figure 4a.**
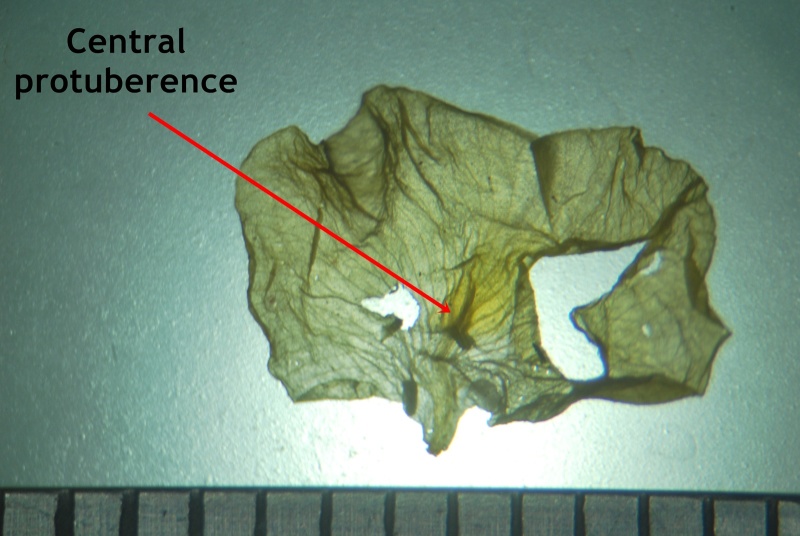
Standard petal

**Figure 4b.**
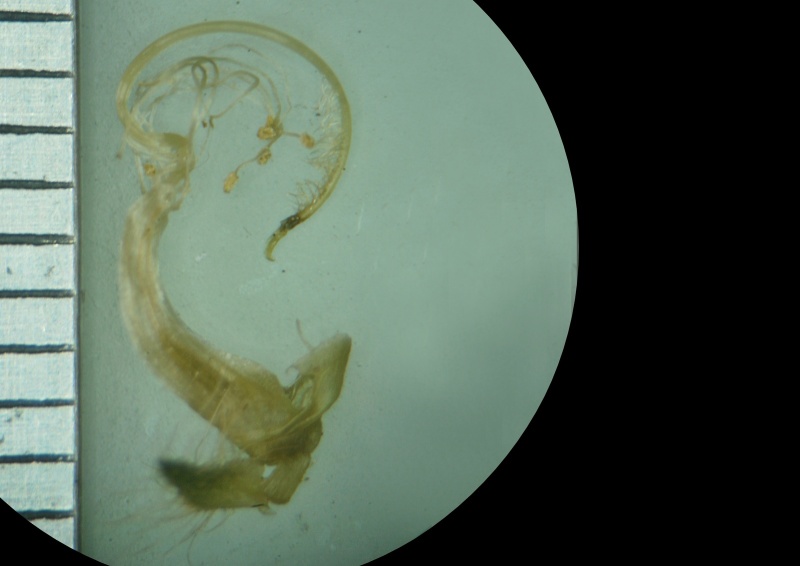
Stamens and pistil

**Figure 4c.**
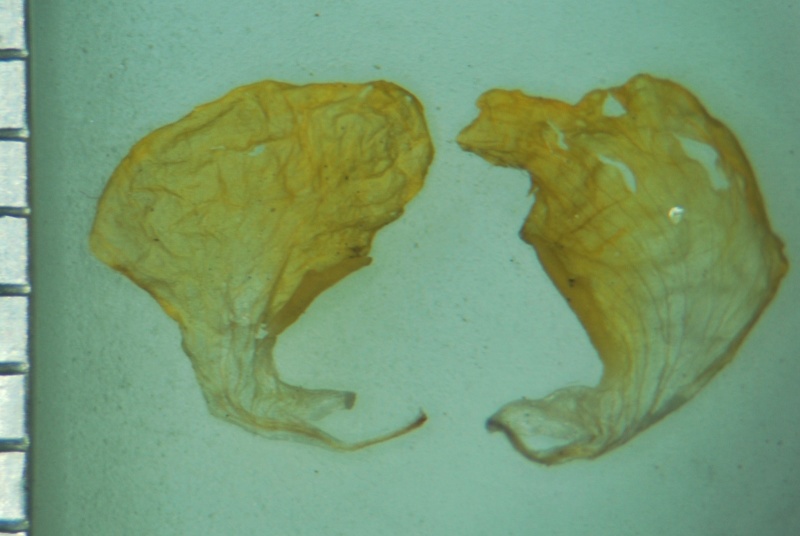
Wing petals

**Figure 4d.**
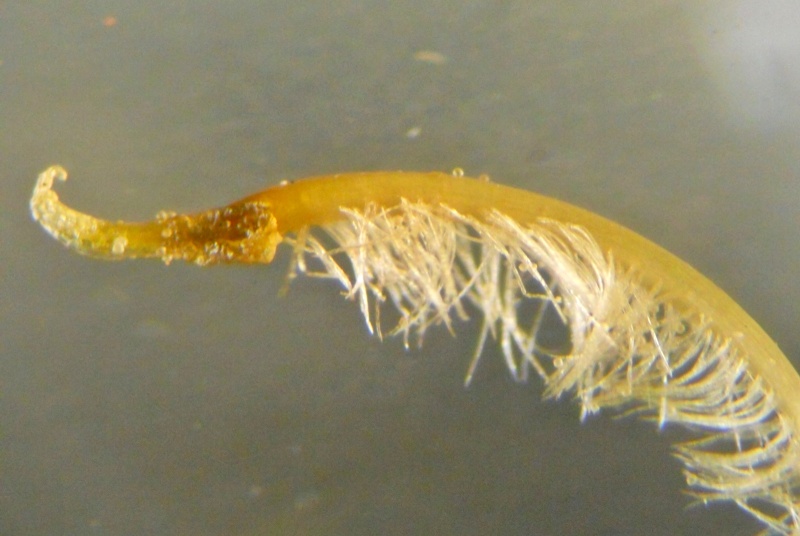
Style beak

**Figure 4e.**
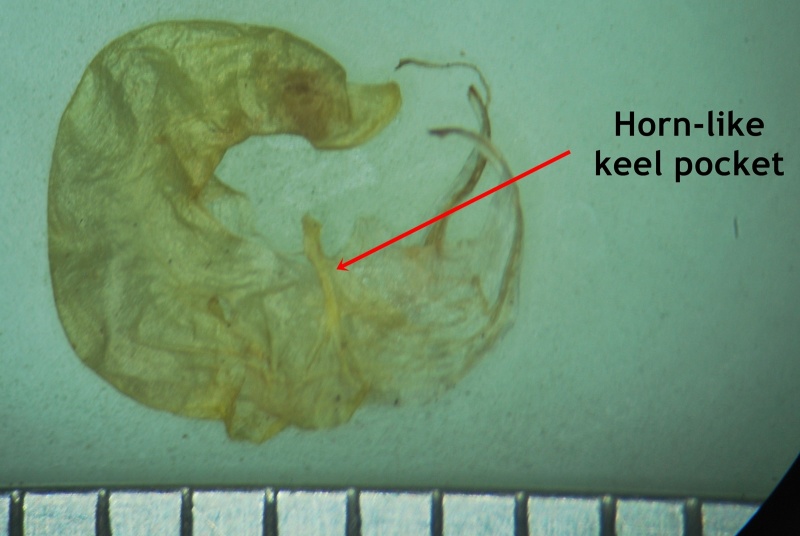
Keel petals

**Figure 4f.**
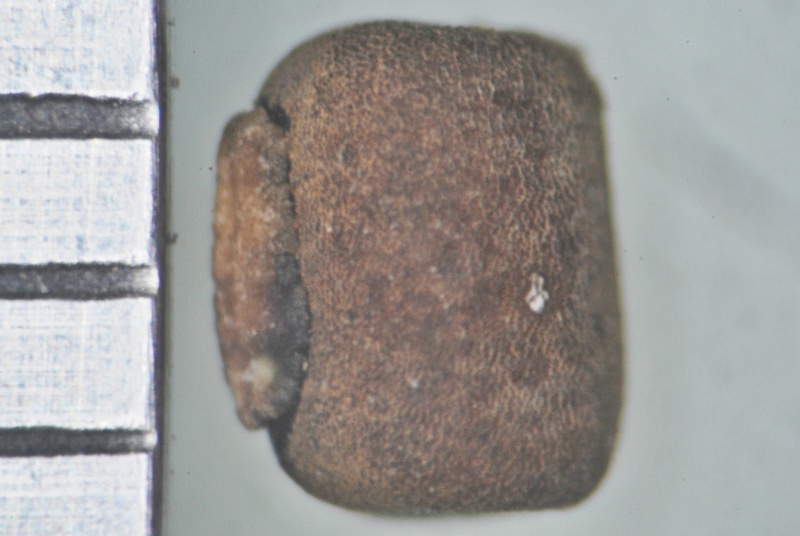
Seed

**Figure 5a.**
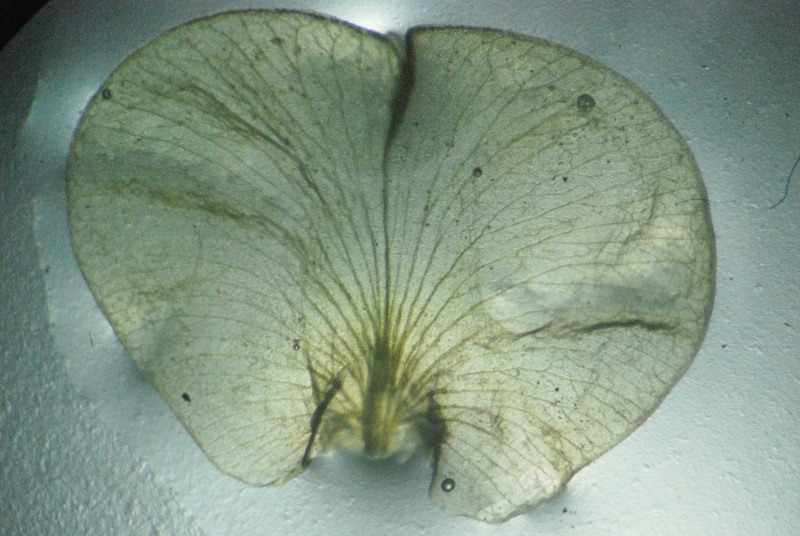
Standard petal

**Figure 5b.**
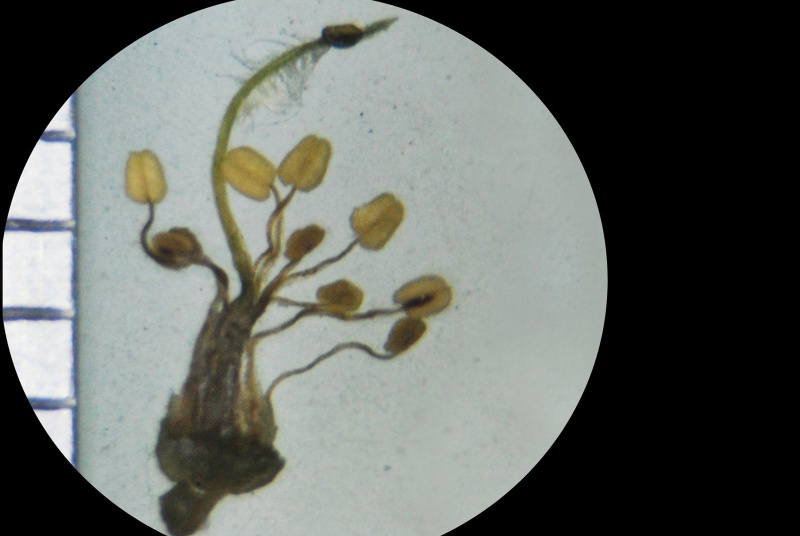
Stamens and pistil

**Figure 5c.**
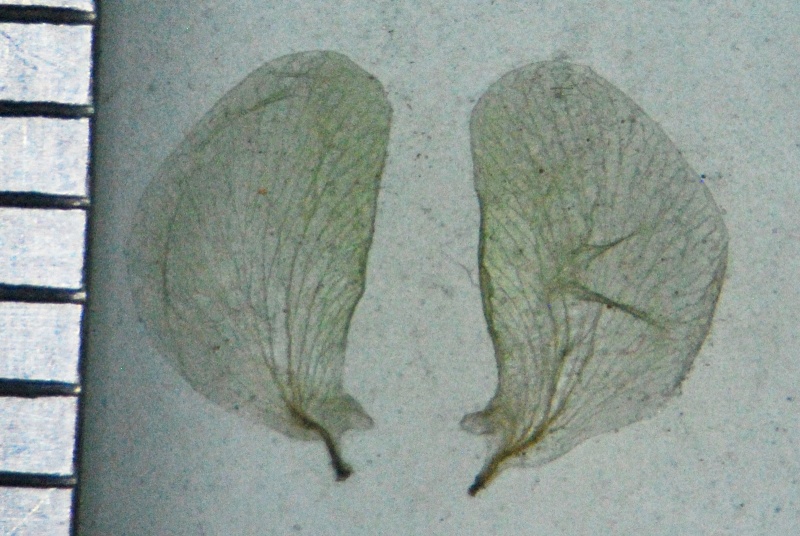
Wing petals

**Figure 5d.**
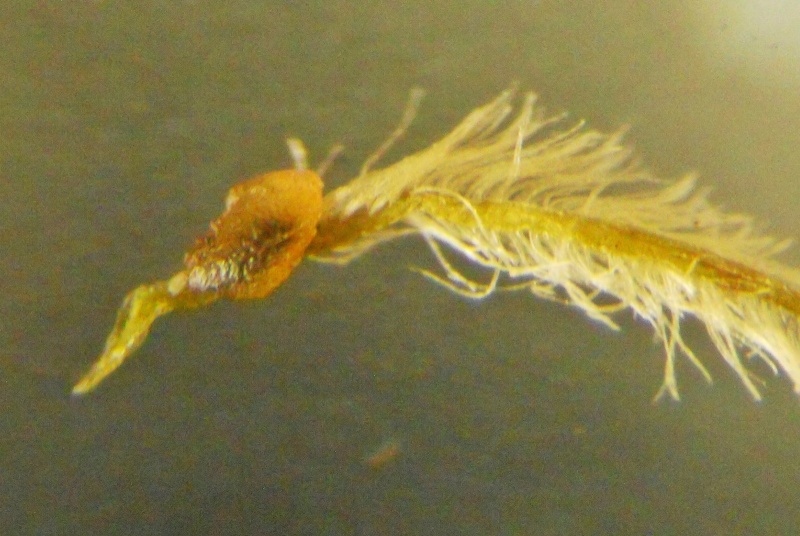
Style beak

**Figure 5e.**
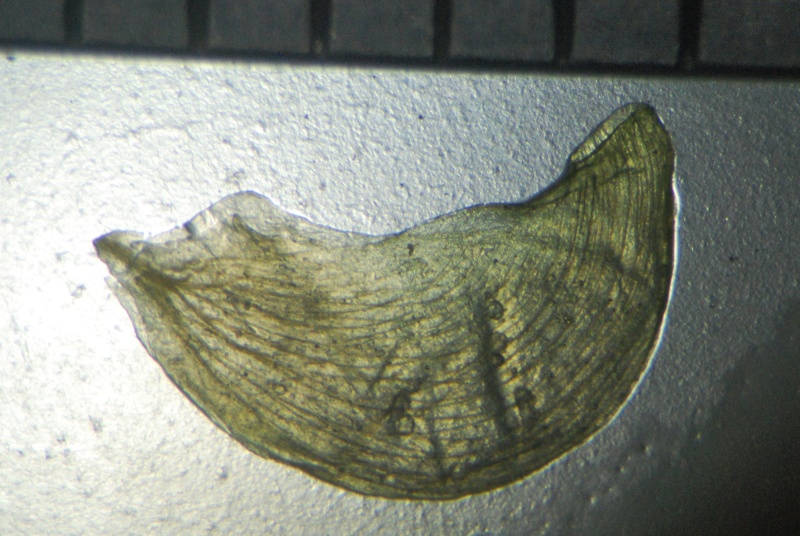
Keel petals

**Figure 5f.**
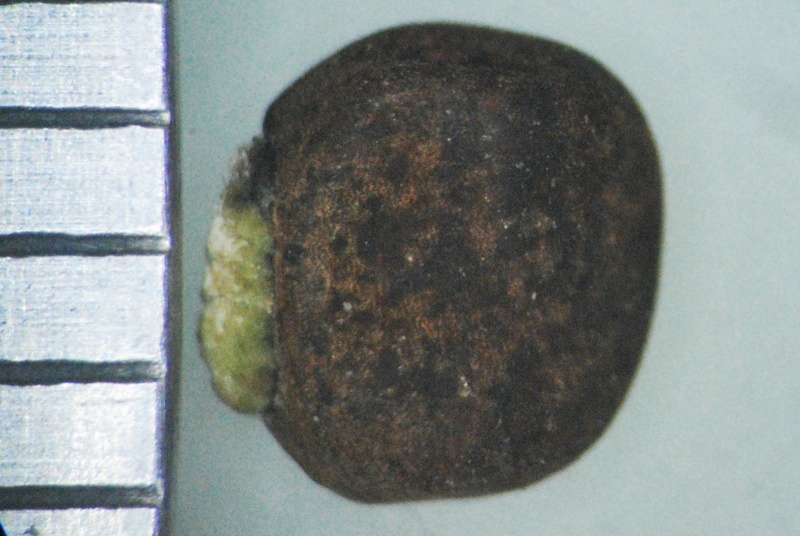
Seed

**Figure 6a.**
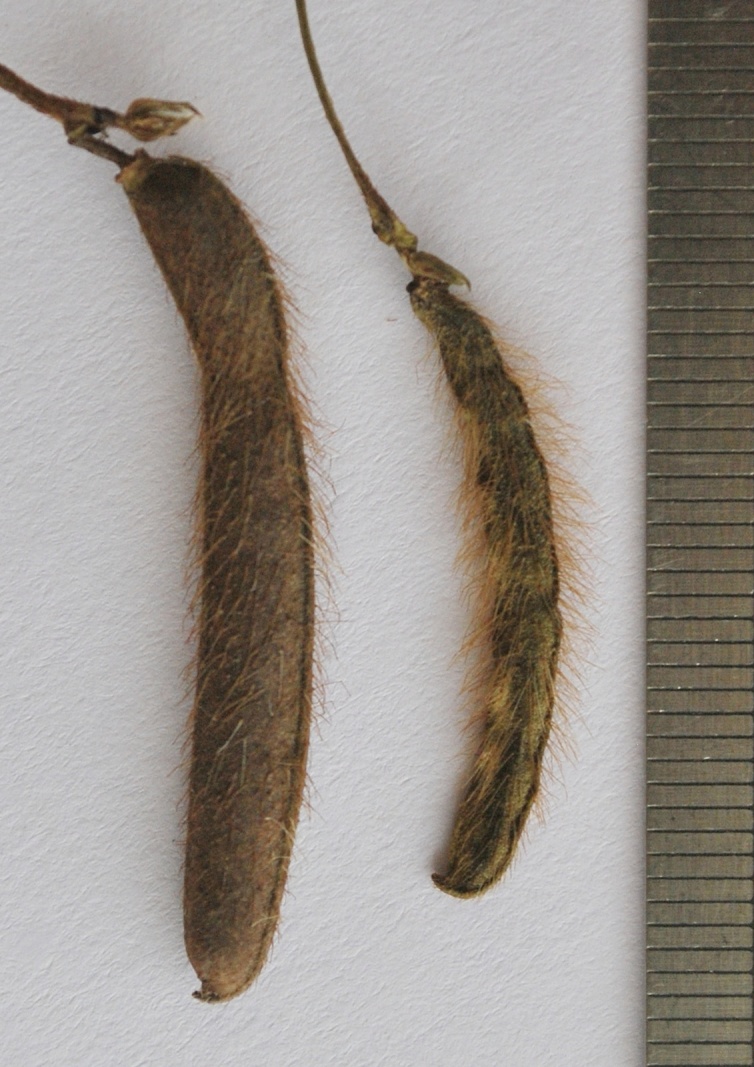
Pods of cleistogamous flower

**Figure 6b.**
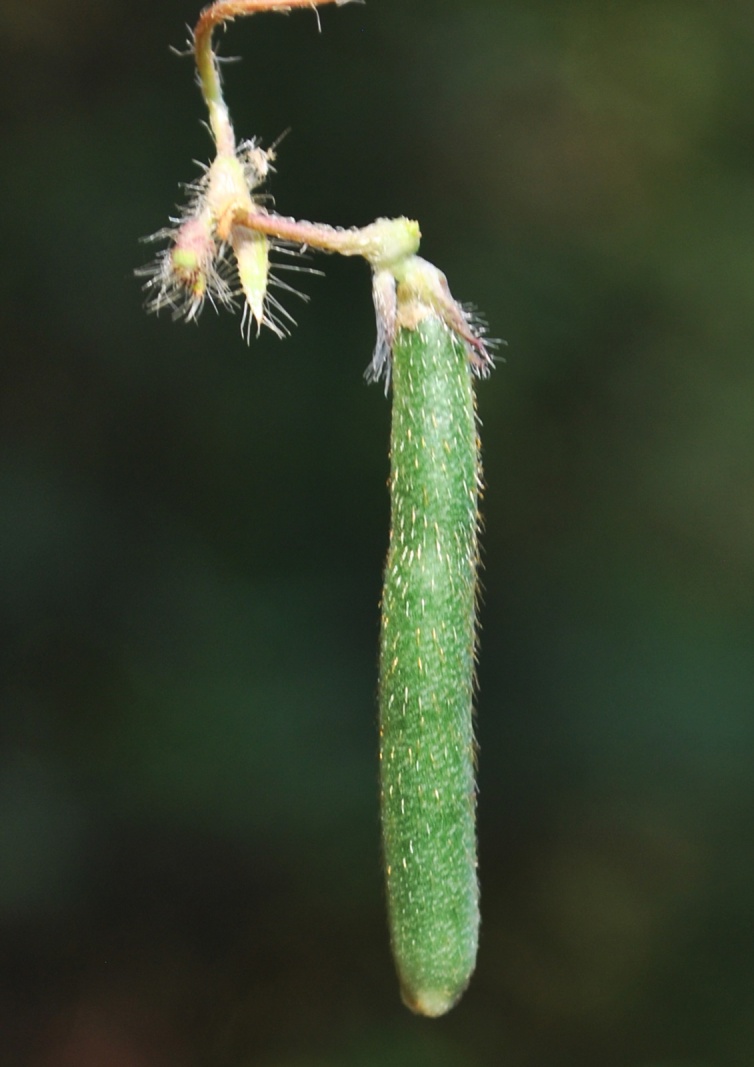
Pod of chasmogamous flower

**Table 1.** Comparison between *Vigna pandeyana* sp. nov., *Vigna yadavii* Gaikwad et al. and *Vigna dalzelliana* (Kuntze) Verdc.

**Characteristics**	***Vigna pandeyana***	***Vigna yadavii***	***Vigna dalzelliana***
*Shoots*	dimorphic (i.e. someone aerial,normal leafy and the others leafless, which produceds atfrom the lower nodes of the stem)	not dimorphic (only normal leafy shoot present)	not dimorphic (only aerinormal leafy shoots present)
*Seed germination*	epigeal	hypogeal	hypogeal
**Cleistogamous flowers**			
*Flowers*	present on leafless subterranean shoots which produced atfrom the lower nodes of the stem, close to soil surface	present on underground positively geotropic branches	Cleistogamous flowers absent
*Central protuberance of standard petal*	absent	present (*ca* 1 mm long)
*Horn-like pocket of keel petal*	absent	present (1.6-2 mm long)
*Style*	curved with 0.3-0.5 mm long beak	‘*S*’ shaped with 0.2-0.3 mm long beak
*Pods*	straight, 2.5-4 cm long, sparsely hairy, green when young and turn brown with maturity	curved, 1.5-2.5 cm long, glabrescent, white/albino
*Seeds*	8-9 per pod, obliquely rounded (3.5-4 x 3-3.5 mm), dark brown	3-5 per pod, oblong or sub-cylindric (2.5-3 x 2-2.2 mm), whitish brown,
*Seed coat*	foveolate with mesh-like reticulation	non-foveolate, shiny
*Hilum*	well developed, 2-2.2 mm long, protruding	poorly developed, 1-1.1 mm long, not protruding
**Chasmogamous flowers**			
*Pods*	4-10 seeded, densely covered with 2-3 mm long brownish hairs	6-12 seeded, sparsely covered with *ca* 0.7 mm long whitish hairs	8-10 seeded, quite glabrous
*Seed coat*	foveolate with mesh-like reticulation	non-foveolate, shiny	foveolate

## References

[B1218954] Aitawade M. M., Sutar SP, Rao SR, Malik SK, Yadav SR, KV Bhat (2012). Section Ceratotropis of subgenus *Ceratotropis* of *Vigna* (Leguminosae - Papilionoideae) in India with a new species from Northern Western Ghats. Rheedea.

[B1218282] Babu C. R., Sharma SK, Johri B. M. (1987). Leguminosae-Papilionoideae: Tribe-Phaseoleae. Bulletin Botanical Survey of India.

[B1218292] Delgado-Salinas A., Thulin M, Pasquet R, Weededn N, Lavin M (2011). *Vigna* (Leguminosae) s.l.: The names and identities of the American segregate genera. American Journal of Botany.

[B1218326] Gaikwad S., Gore R, Randive S, Garad K (2014). *Vigna
yadavii* (Leguminosae: Papilionoideae), a new species from Western Ghats, India. Biodiversity Data Journal.

[B1218365] Lewis G. P., Shrine B, Mackinder B, Lock J. M. (2005). *Legumes of the World*.

[B1218374] Maréchal R., Mascherpa JM, Stainier F (1978). Etude taxonomique d’un groupe complex d’espécies des genres *Phaseolus* et *Vigna* (Papilionaceae) sur la base de données morphologiques et polliniques, traitées par I’anyse informatique. Boissiera.

[B1218384] Maxted N., Mabuza-Dalamini P, Moss H, Padulosis S, Jarvis A, Gaurino L (2004). *African Vigna: Systematic and Ecogeographic studies*.

[B1218395] Tateishi Y (1984). Contribution to the genus *Vigna* (Leguminosae). Science Report, Tohoku University. Series 4 (Biology).

[B1218405] Thulin M., Lavin M, Pasquet R, Delgado-Salinas A (2004). Phylogeny and Biogeography of *Wajira* (Leguminosae): a Monophyletic segregate of *Vigna* centered in the horn of Africa region. Journal of Systematic Botany.

[B1218428] Tomooka N., Maxted N, Thavarasook C, Jayasuriya A. H.M. (2002). Two new species, sectional designations and new combinations in Vigna
subgenus
Ceratotropis (Piper) Vedc. (Leguminosae: Phaseoleae). Kew Bulletin.

[B1218451] Tomooka N., Vaughan D, Moss H, Maxted N (2002). *The Asian Vigna: Genus Vigna subgenus Ceratotropis genetic resources*.

[B1218460] Toomoka N., Kaga A, Isemura T, Vaughan U, Kole C (2010). Vigna. *Wild Crop Relatives: Genomic and Breeding Resources, Legume Crop and Forages*.

